# Genetic Advances in *Cannabis sativa* L.: A Review of Recent Progress and Future Directions

**DOI:** 10.3390/plants15132088

**Published:** 2026-07-04

**Authors:** Kasuni C. Daundasekara, Kalpani P. Thennakoon, Jivendra S. Wickramasinghe, Selamawit Woldesenbet, Christopher Delhom, Suman Chandra, Aruna D. Weerasooriya

**Affiliations:** 1 CAFNR Research, College of Agriculture, Food and Natural Resources, Prairie View A&M University, Prairie View, TX 77446, USA; kthennakoon@pvamu.edu (K.P.T.); jswickramasinghe@pvamu.edu (J.S.W.); sewoldesenbet@pvamu.edu (S.W.); 2 Cotton Ginning Research Unit, USDA Agricultural Research Service, Stoneville, MS 38756, USA; chris.delhom@usda.gov; 3 National Center for Natural Products Research, School of Pharmacy, The University of Mississippi, University, MS 38677, USA; suman@olemiss.edu; 4 Department of Biomolecular Sciences, School of Pharmacy, The University of Mississippi, University, MS 38677, USA

**Keywords:** *Cannabis sativa* L., industrial hemp genomics, molecular breeding, functional genomics, trait genetics, genome engineering

## Abstract

*Cannabis sativa* L. is an economically significant multi-use crop valued for fiber, seed, and phytochemical production. Compared with other crops, advancement in *Cannabis sativa* has been slow due to regulatory constraints and genetic resource limitations. Recent advances in technology have transformed the research landscape, supporting a deeper understanding of the genetic architecture underlying key agronomic traits. This review summarizes current progress in *Cannabis sativa* genetics and genomics, mainly focusing on structural genome organization, including chromosome-level assemblies and emerging pangenomic resources that capture species-wide diversity. We explore the molecular basis of key agronomic traits, including sex determination, cannabinoid biosynthesis, fiber quality, seed composition, disease resistance, and abiotic stress tolerance, highlighting their complex regulatory networks. Functional genomics tools including virus-induced gene silencing, transient expression systems, and CRISPR/Cas9 genome editing are reviewed as approaches enabling direct gene functional validation. We further review integration of these resources with molecular breeding strategies, including marker-assisted and genomic selection, to accelerate elite genotype development. Finally, we address persistent challenges such as genomic complexity, reference bias, and phenotyping limitations while outlining future research directions. Together, these advances position *C. sativa* as a compelling system for both fundamental plant biology and applied crop improvement.

## 1. Introduction

*Cannabis sativa* L. is a versatile, multi-purpose crop with considerable genetic diversity, broadly divided into distinct groups, such as industrial hemp, usually defined by containing ≤0.3% Δ9-tetrahydrocannabinol (THC) and narcotic marijuana genotypes characterized by higher THC content [1,2]. The significance of this crop has increased considerably due to its wide range of industrial applications and its uses in human nutrition and medicinal products [3,4,5]. The origin of the species trace back to eastern Asia [6,7]. For thousands of years, *Cannabis sativa* has been cultivated for its fiber, oil-rich seeds, and other valuable compounds [8,9]. In the United States, interest in industrial hemp cultivation increased following the 2018 legalization of low-THC varieties [2,10], and hemp is now recognized as a valuable agricultural commodity [11,12,13]. Compared with marijuana, industrial hemp contains smaller amounts of THC and is grown mainly for its cannabidiol (CBD) content, as well as for fiber and grain production [3,14]. This renewed interest coincides with rapid advances in plant genomics, molecular biology, and breeding technologies, making it a timely model for modern crop improvement systems.

Despite its long history of use, genetics and breeding research in industrial hemp has lagged behind that of other major crops. For most of the past century, legal restrictions on *Cannabis* in many regions curtailed scientific research and limited the exchange of genetic resources across borders [15,16]. As a result, assessments of genetic variation were historically confined to phenotypic selection, with little to no molecular data being generated [17,18]. The reclassification and legalization of industrial hemp in several regions, such as under the 2018 U.S. Farm Bill, have transformed the research landscape [2]. Advances in next-generation sequencing, coupled with international efforts to develop reference genomes, have provided unprecedented insights into the biology and evolution of industrial hemp [19,20,21,22], making it possible to dissect complex trait variations at very small scales, analyze genome-wide diversity, and accelerate the use of marker-assisted and genomic selection methods.

**Figure 1:**
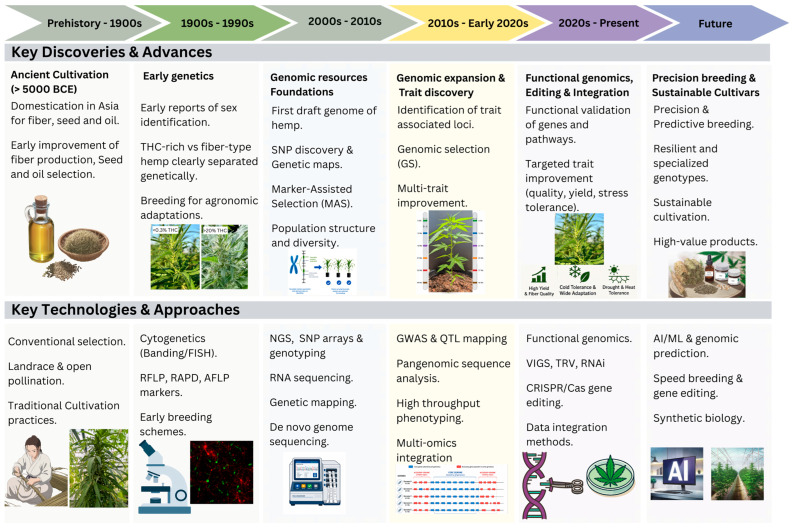
Historical progression of genetic and genomic advances in *Cannabis sativa* L. breeding and research. Schematic timeline showing major milestones in *Cannabis sativa* genetic improvement, from early cultivation to modern precision breeding. The upper panel summarizes key breeding objectives and genetic advances across successive eras, including the differentiation of low-THC industrial hemp (<0.3% THC) from drug-type *Cannabis*, the adoption of marker-assisted selection (MAS), and the development of climate-resilient cultivars. The lower panel illustrates the progression of breeding technologies, from cytogenetics and next-generation sequencing (NGS) to pangenomic analysis, multi-omics integration, CRISPR/Cas-based genome editing, and AI/ML-assisted genomic prediction. The figure was assembled in Canva, with selected illustrations generated using the DeepAI image generation tool.

Recent progress in plant molecular biology, genomics, and biotechnology has greatly deepened our understanding of the complex genetic architecture underlying important agronomic traits in *Cannabis sativa* (Figure 1). This has allowed for increased precision in the improvement of those traits through both traditional breeding and emerging genetic engineering strategies [23,24,25]. However, industrial hemp has a number of distinctive biological challenges for genetic improvement (i.e., dioecy, high heterozygosity, broad levels of genetic diversity and limited self-pollination capacity) that can complicate controlled breeding, functional confirmation and development of stable genotypes [26,27,28].

In recent years, researchers have published several review articles on *Cannabis sativa*, exploring from how the plant is classified to how it is grown and used. The early reviews on *Cannabis sativa* formed a basis for research on botanical characteristics, classification, reproductive biology, systematics, and genetic diversity, which laid the foundation for understanding the origins and diversity of this plant [9,29]. Many researchers have also explored the industrial side of *Cannabis sativa*, focusing on its fiber and seed applications [30,31,32]. Industrial hemp fiber composition, structure, extraction, processing methods, and applications in textiles have been widely discussed. Industrial hemp seed proteins, essential fatty acids, fiber, and bioactive chemicals are being thoroughly studied because it is thought to be a very nutritious seed [4,33,34,35]. In addition, a few reviews have explored environmental applications such as phytoremediation and circular bioeconomy potential [36,37]. Research on industrial sustainability has looked at hemp as a renewable crop, looking at the economic, environmental, and social aspects [3,10,38,39].

Recent studies focus more on the genetic and genomic improvement of *Cannabis sativa*. These reviews address molecular markers, sequencing resources, genome assemblies, and functional genomics approaches [40]. They discuss how this helps advance our understanding of genetic diversity, cannabinoid biosynthesis, sex determination, and complex trait regulation [41,42,43]. Some researchers have also explored challenges of genome-editing technologies and breeding innovations in industrial hemp improvement [26,44,45].

Despite this growing body of work, existing reviews still vary considerably in their scope and depth of integration. Agronomic and industrial studies mostly concentrate on production and practical use, while genomic and molecular reviews highly lean toward gene discovery and sequencing resources, leaving a gap between their findings and how those findings guide breeding practices. This gap emphasizes the need for a more thorough discussion that brings together phenotypic characterization, multi-omics data, genetic mapping, genomic prediction, and breeding strategies under one framework.

This review addresses this gap by providing an integrated framework for *Cannabis sativa* improvement. We integrate phenotypic and molecular data with modern breeding approaches, emphasizing how these data streams contribute to breeding through functional understanding, predictive modeling, and the development of improved cultivars. We discuss recent advances in the genetics, genomics and multi-omics approaches of *Cannabis sativa* research, with a focus on genome-sequencing efforts, gene function characterization, molecular marker development, and genetic transformation methodologies. We discuss the new technologies that are changing the way we think about improving *Cannabis*, such as CRISPR/Cas gene editing, transient expression systems, and RNA interference technologies. By integrating these advances, we aim to provide a comprehensive overview of how modern genetic tools are transforming *Cannabis* research and enabling the development of elite genotypes, optimized for traits including fiber quality, seed composition, phytochemical profiles, and resilience to environmental stressors.

## 2. Genome Architecture and Evolution of *Cannabis sativa*

The latest findings in the comprehension and analysis of *Cannabis sativa* genomics have increased our understanding of how genome structure shapes evolution and phenotypic variation. The chromosomal arrangement and overall genomic organization of *Cannabis* provide valuable context for interpreting patterns of trait and population variation, as well as the genetic architecture of key agronomic traits.

**Figure 2:**
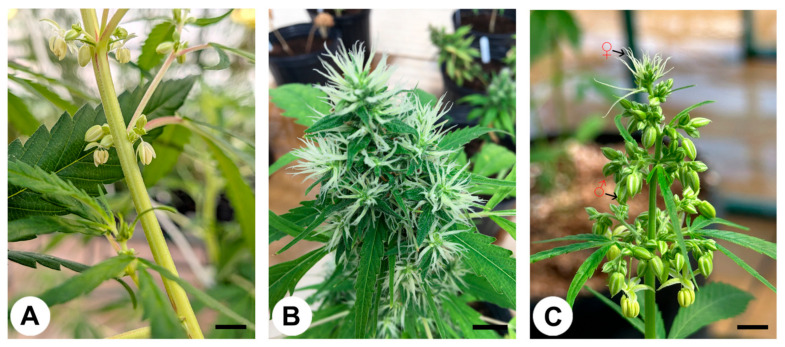
Floral morphology and sex expression in industrial hemp (*Cannabis sativa* L.). (**A**) Male plant showing early staminate inflorescences with developing pollen sacs along the nodes. (**B**) Female plant at the flowering stage with compact pistillate inflorescences and trichome-rich floral bracts. (**C**) Monoecious plant bearing both male (♂) and female (♀) flowers on the same individual. Scale bar = 1 cm. Photographs were taken by the authors, and the figure was assembled using Adobe Photoshop.

*Cannabis sativa* L. is a diploid species (2*n* = 20) that has nine pairs of autosomes and one pair of heteromorphic sex chromosomes [19,46]. The species is predominantly dioecious (one sex per plant), though some plants can be monoecious (bearing both male and female flowers) (Figure 2) [47]. Sex determination is genetically controlled, with females carrying XX chromosomes and males carrying XY chromosomes [48,49]. Previous studies reported similar diploid genome sizes between sexes. Approximately 1636 (±7.2) Mb for females and 1683 (±13.9) Mb for males [27,50,51]. This difference can largely be attributed to the presence of the larger Y chromosome in males [51].

One of the most defining features of the *Cannabis* genome is having a high quantity of repetitive DNA. The majority of *Cannabis*’ repetitive DNA comes from long terminal repeat (LTR) retrotransposons, which contribute to more than 60% of the overall *Cannabis* genome [52,53,54]. These repetitive elements contribute to overall genome size expansion and offer insights into gene location and the evolutionary history of the *Cannabis* genome [52]. The accumulation, especially within heterochromatic regions and in the genomic regions surrounding the sex chromosomes, has been attributed to the increased size and structural variation observed between male and female *Cannabis* plants [55].

The presence of repetitive sequences in a genome challenges for accurate genome assembly when short-read sequencing methods are used [52]. However, advances in sequencing using long reads and chromatin conformation capture methods enabled assembly of genomic sequences (Table S1) that contain many repetitive sequences and clarify genome organization [19,56]. This structural knowledge is important when interpreting recombination patterns, improving the resolution of trait mapping, and understanding the structural variation associated with agronomic or phenotypic traits.

## 3. Reference Genomes, Assemblies, and Pangenomics

Over the past decade, genomic resources for *Cannabis sativa* have advanced considerably, yielding deeper insights into cannabinoid biosynthesis, chemotype determination, and trait evolution. The first draft genome of *Cannabis sativa* was published in 2011, based on a drug-type cultivar known as Purple Kush. This allowed for the completion of the first genome and transcriptome assemblies and the opportunity to conduct comparative analyses of traditional fiber-type genotypes while also establishing the foundation for molecular studies of cannabinoid biosynthesis [46]. Building on this initial draft genome assembly, researchers were able to create high-quality chromosome-level assemblies that clarified the complex organization of cannabinoid synthase loci, providing critical structural insights into how tetrahydrocannabinolic acid (THCA) and cannabidiolic acid (CBDA) are biosynthesized [57,58].

Following the Purple Kush assembly, additional assemblies of other high CBD and wild *Cannabis* genotypes further expanded the understanding of gene content, repetitive elements and genome organization, supporting both functional genomics and molecular breeding opportunities [19,56]. These genomic resources also identified regions of the *Cannabis* genome with variable and conserved gene sequences relating to cannabinoid production, fatty acid metabolism and vitamin E biosynthesis, serving as a broad-purpose reference for studying agronomic traits and chemotype diversity.

Haplotype-resolved assemblies and large-scale pangenomic analyses using numerous accessions demonstrated that *Cannabis* harbors extensive genotypic diversity, with much of it being driven by gene presence/absence variation and patterns of gene exchange among three major population groups, industrial hemp, drug-type, and basal [20,21]. The basal group includes Chinese landraces and feral accessions that diverged prior to modern industrial hemp and drug-type genotypes and retain ancestral genetic variation. These provide insights into the domestication and evolutionary history of *Cannabis* [20,59]. Stress and disease-resistant genes were found to be overrepresented in flexible genes, indicating the potential for adaptation and breeding applications [20]. Pangenomic frameworks therefore offer an improved basis for mapping traits, reducing reference bias and enhancing the development of marker for breeding.

In addition to genome-wide and pangenomic approaches, short tandem repeats (STRs) have been used to individualize *Cannabis* plants and determine their origin for forensic purposes [60]. STR profiling systems have undergone internal validations and have demonstrated the ability to differentiate among individual plants, associate plant material with seized samples, and detect clonal propagation [61,62].

More recently, haplotype-resolved genome assemblies of seed-type *Cannabis sativa* have added further dimension to the understanding of genome organization beyond the reference genome and pangenome paradigm [63]. This allows for the distinction between maternal and paternal alleles and revealing genetic differences that remained concealed before and enhancing structural accuracy in highly heterozygous genomic loci [63]. In summary, these genomic advances define a strong foundation for understanding cannabinoid pathways, genome evolution, and accelerating the enhancement of industrial hemp genotypes for use in fiber and seed production and secondary metabolites, while also supporting high-resolution genetic identification and forensic applications.

### Plastid Genome Diversity in Cannabis sativa

Plastid genome analyses have provided an additional layer of genomic information in *Cannabis sativa*, complementing nuclear genome studies of diversity and evolution. The complete chloroplast genome sequencing of multiple genotypes, including *Cannabis sativa* var. Yunma 7, has enabled detailed characterization of plastome structure, gene content, and evolutionary conservation [64,65]. Comparative analyses extending to related taxa, such as *Humulus lupulus*, further highlight the high structural conservation of chloroplast genomes within the Cannabaceae family while still allowing for discrimination among distinct lineages [66].

In addition to whole-plastome sequencing, chloroplast-derived SNP markers have been developed and assessed for crop-type determination and biogeographical origin tracing in European *Cannabis sativa* samples, demonstrating the value of plastid variation for forensic and breeding applications [67]. Additionally, integrated genomic databases combining nuclear, chloroplast, and mitochondrial data from U.S. *Cannabis* accessions have expanded reference resources for multi-genome comparisons, enabling more robust phylogeographic and genotype-level analyses [68]. Comparative plastome studies consistently indicate that while the chloroplast genome is relatively conserved, it contains sufficient variation in SNPs and simple sequence repeats (SSRs) to support genotype identification, population structure inference, and germplasm traceability [69]. Together, these organelle genome resources complement nuclear genome-based approaches and strengthen the genomic toolkit available for industrial hemp breeding and evolutionary research.

## 4. Sex Determination and Reproductive Biology

Sex determination is one of the most significant aspects of *Cannabis sativa* biology, as it affects not only reproductive development but also many other traits with agronomy and commercial importance. To produce industrial hemp crops with controlled sexual expression, it is important to understand the genetic and molecular basis of sex differentiation for both evolutionary and applied breeding programs.

Unlike many crop species, *Cannabis* is predominantly dioecious, producing male and female flowers on separate individuals. Female plants are generally preferred for cannabinoid production, as unfertilized female inflorescences accumulate substantially greater concentrations of cannabinoids and other specialized metabolites. As a result, understanding the genetic mechanisms regulating sex determination is critical to both biological research and commercial cultivation. From a chromosomal perspective, *Cannabis sativa* follows an XY/XX sex determination system, with males carrying XY chromosomes and females carrying XX [70,71]. However, naturally occurring monoecious individuals are frequently employed in the breeding of fiber and seed industrial hemp [72,73].

Early cytogenetic studies with fluorescence in situ hybridization (FISH) using DAPI/C banding provided initial evidence of heteromorphic sex chromosomes in *Cannabis*, laying the groundwork for subsequent molecular investigations into sex determination [70]. In dioecious *Cannabis sativa*, the sex chromosomes are highly heteromorphic. The X chromosome is among the largest in the karyotype, while the Y chromosome is distinguished by dense accumulation of heterochromatin and repetitive DNA sequences. The lack of recombination outside the pseudo autosomal region suggests that these chromosomes represent an early stage of evolutionary divergence [70,71]. Generally, monoecious cultivars carry an XX karyotype, implying that the development of bisexual flowers arises from differential regulation of sexual expression rather than simply from the presence of a Y chromosome [47].

Although the chromosomal basis of sex determination is well established, the identification of the primary genetic regulators remains an ongoing area of active research. Recent genomic studies have uncovered an ancient locus on the X chromosome carrying three candidate genes responsible for sexual development in *Cannabis sativa*, offering new insights into the mechanisms of sex determination and substantiating the idea that sex determination in *Cannabis* is linked to ancestral elements on the X chromosome [74].

Sex determination in *C. sativa* extends well beyond simple chromosomal inheritance. It is shaped by a broader network of polygenic and regulatory interactions. Genome-wide association studies and resequencing analyses have mapped sex-associated quantitative trait loci (QTLs) across both sex chromosomes and autosomes, linking genes involved in auxin and gibberellin signaling, as well as transcription factors that guide floral development [75,76]. Some of these QTLs overlap with flowering time loci, suggesting that reproductive timing and sexual differentiation are likely co-regulated. Transcriptomic analyses have revealed extensive sex-biased gene expression occurring before visible floral differentiation, implicating both sex-linked and autosomal genes and highlighting early molecular divergence between male and female developmental programs [76]. Recent haplotype-resolved genome assemblies further strengthened existing theories about X Y divergence because they provide direct proof of the structural differences and repeat elements that have accumulated in the Y chromosome over time [63].

These genetic and molecular mechanisms have direct implications for reproductive biology. Sexual phenotype determines flower morphology, the timing of anthesis, and the production of pollen and seeds, all of which influence both natural population dynamics and cultivated breeding systems. Inflorescence architecture is an important aspect of *Cannabis* reproductive biology that shapes pollination efficiency, cannabinoid production, disease susceptibility, and seed retention [77,78,79,80]. Female inflorescences develop acropetally, with flowers initiating and maturing progressively from base to apex [80,81,82]. Drug-type cultivars typically form compact inflorescences with short internodes and dense floral clusters, while industrial hemp cultivars tend toward longer internodes and a more open structure [83,84]. The genetic mechanisms governing inflorescence development, floral density, and branching architecture remain poorly understood and represent promising targets for future research [78,79].

Environmental factors and phytohormones also play a role in modulating sex expression. For instance, exogenous application of ethylene inhibitors such as silver thiosulfate can induce male flower development on genetically female plants, a technique widely used to produce feminized seed [76]. These insights have translated into practical tools for early sex identification, a capability of particular value in cannabinoid-focused breeding, where female plants are commercially preferred. Y-linked PCR markers and multiplex assays have proven capable of reliably distinguishing male from female seedlings across a range of genotypes, though their accuracy can vary depending on germplasm background [85,86]. One of the most recent advances in this direction refers to CRISP, an affordable and scalable tool for early sex determination in *Cannabis sativa*. CRISP offers a rapid and cost-effective pipeline for sex identification, with the potential to meaningfully improve the efficiency of early sex determination in industrial hemp breeding programs [87]. Integrating these tools with an understanding of polygenic regulation and hormonal influences would give breeders meaningful control over sex expression, with direct effects on reproductive efficiency and cannabinoid biosynthesis.

Sex determination in *C. sativa* is a far more complex process than once thought, operating through a multilayered system that integrates chromosomal architecture, polygenic regulation, and environmental responsiveness. This integrated framework not only shapes reproductive phenotypes central to cultivation and breeding but also reflects the ongoing evolution of sex chromosomes and regulatory networks in the species. Despite significant progress, several fundamental questions regarding *Cannabis* sex determination remain unresolved. The primary genetic regulators of sex determination have yet to be definitively identified, the interactions between chromosomal and hormonal pathways remain poorly understood, and functional validation of candidate genes is limited. Moving forward, research that brings together cytogenetic, genomic, and transcriptomic approaches will be essential both to resolving the full molecular architecture of sex determination and to translating that knowledge into more effective breeding strategies and a better understanding of how sex chromosomes evolve in plants.

## 5. Genetics of Cannabinoid Biosynthesis

Cannabinoids are a chemically diverse class of terpenophenolic compounds unique to *Cannabis sativa* and have been extensively studied. They represent one of the most economically important trait groups in the species. Over one hundred cannabinoids have been identified, with Δ9-tetrahydrocannabinol (THC) and cannabidiol (CBD) being the most broadly studied due to their psychoactive and therapeutic properties, respectively [88,89]. The high variation in cannabinoid profiles across industrial hemp and drug-type cultivars arises from complex interactions among biosynthetic genes (Table 1), structural genomic variation, transcriptional regulation, and environmental influences. The blend of cannabinoids a plant produces defines its chemotype, and this chemical identity highlights the plant’s industrial, medicinal, and recreational applications [90,91,92].

**Figure 3:**
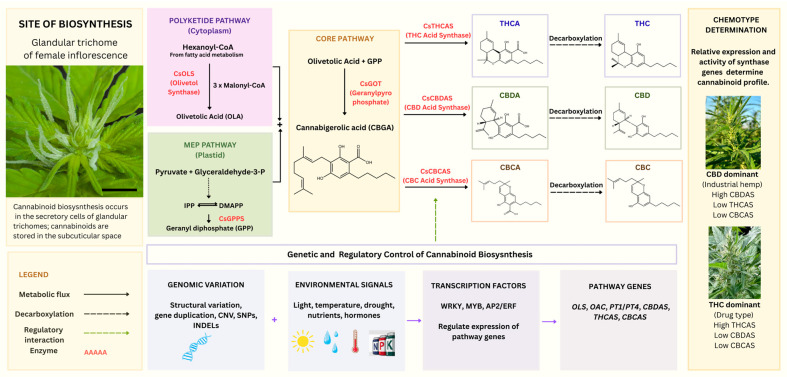
Biosynthetic pathway and regulatory network of cannabinoids in *Cannabis sativa* L. Cannabinoid biosynthesis occurs in the secretory cells of glandular trichomes on female flowers. The pathway begins with two parallel routes: the cytosolic polyketide pathway, in which hexanoyl-CoA and malonyl-CoA are converted to olivetolic acid (OLA) by olivetol synthase (CsOLS), and the plastidial MEP pathway, which converts pyruvate and glyceraldehyde-3-phosphate into isopentenyl diphosphate (IPP) and dimethylallyl diphosphate (DMAPP). These intermediates are condensed by geranyl diphosphate synthase (CsGPPS) to produce geranyl diphosphate (GPP). OLA and GPP are then combined by the prenyltransferase CsGOT to form cannabigerolic acid (CBGA), the common precursor of major cannabinoids. CBGA is subsequently converted into tetrahydrocannabinolic acid (THCA), cannabidiolic acid (CBDA), and cannabichromenic acid (CBCA) by CsTHCAS, CsCBDAS, and CsCBCAS, respectively. These acidic cannabinoids are decarboxylated by heat or light to produce the neutral cannabinoids THC, CBD, and CBC. Cannabinoid biosynthesis is regulated by genetic variation, environmental conditions, and transcription factor families, including WRKY, MYB, and AP2/ERF. Scale bar = 5 mm. The figure was designed and assembled in Canva (v1.122.0.0), and the chemical structures were generated using the RCSB Chemical Component Sketch Tool.

The glandular trichomes of female inflorescence serve as the center of the production of cannabinoids, reflecting a highly spatially organized process (Figure 3), where it integrates metabolic flux from both the polyketide and plastidial methylerythritol phosphate (MEP) pathways. The biosynthetic pathway is initiated by the production of olivetolic acid through a type III polyketide synthase, followed by prenylation to form cannabigerolic acid (CBGA), from which the major cannabinoids are derived [93,104,105].

From CBGA, a set of specialized oxidocyclases catalyze the formation of the major cannabinoid acids, tetrahydrocannabinolic acid (THCA), cannabidiolic acid (CBDA), and cannabichromenic acid (CBCA). The relative activity of these enzymes is what largely determines a plant’s chemotype. These enzymes constitute the core genetic determinants of cannabinoid composition and have been major targets of both genetic and breeding investigations. In industrial hemp, CBDAS activity dominates over that of THCAS and CBCAS, steering the plant toward high CBDA accumulation [93,94,105].

The genome-wide inventory and functional analysis of cannabinoid oxidocyclases in *Cannabis sativa* have provided more details about the diversity within cannabinoid synthase gene families, including expansion, sequence divergence, and functional diversification of THCAS, CBDAS, and related enzymes. These findings have deepened our understanding of how chemotypes are determined and how cannabinoid biosynthetic gene families have evolved over time [106]. Recent genomic studies have demonstrated that cannabinoid synthase genes occur within highly dynamic genomic regions characterized by tandem duplications, deletions, and structural rearrangements [107]. Copy number variation within THCAS and CBDAS clusters has been associated with differences in cannabinoid profiles among cultivars, suggesting that structural variation contributes substantially to chemotype diversity [108,109].

Transcriptomic research has further revealed that the expression of pathway genes is carefully synchronized with trichome development and that this expression responds dynamically to environmental signals, allowing the plant to adjust cannabinoid output in response to both developmental stage and external stress [104,110,111,112]. Functional experiments, such as overexpression of *CsCBDAS*, demonstrate cross-talk between terminal synthases and genes further upstream in the pathway, highlighting multi-level regulation that fine-tunes cannabinoid biosynthesis [105].

Beyond enzyme level control, cannabinoid biosynthesis is strongly influenced by transcriptional and epigenetic regulation. The responsiveness of gene promoters to phytohormones and environmental stimuli, together with the activity of transcription factor families such as WRKY, AP2L, MYB, and COL, coordinates gene expression and precursor allocation [104,111,112,113]. Together, these mechanisms ensure robust CBDA accumulation and minimal THCA in industrial hemp genotypes while also identifying key regulatory points that can be targeted through metabolic engineering and precision breeding to tailor cannabinoid profiles for fiber, seed, and therapeutic end uses [93,105,113].

Genome-wide association studies and population structure analyses have identified specific genomic loci that underline chemical diversity in the species, revealing how population stratification shapes metabolite variation. This has strengthened the link between genotypic variation and the cannabinoid and terpene biosynthetic pathways in *Cannabis sativa* [22]. Cannabinoid biosynthesis in *C. sativa* is not a simple, linear pathway. It is an interconnected system in which metabolic flux, enzyme specialization, and transcriptional regulation function together. Understanding how these elements interact and how they respond to genetic and environmental variation will be essential to developing more predictable, targeted approaches to cannabinoid production, whether the goal is maximizing therapeutic compounds, meeting regulatory thresholds, or adapting performance across variable growing conditions. Such knowledge will provide a foundation for breeding and developing cultivars tailored to specific commercial and industrial applications.

## 6. Genetic Basis of Fiber Quality and Quantity in *Cannabis sativa*

Fiber production has historically been one of the primary agricultural uses of *Cannabis sativa* and yet the genetic mechanisms controlling fiber development remain considerably more poorly understood than those governing cannabinoid biosynthesis. Fiber quality and yield in *Cannabis sativa* are complex quantitative traits governed by the biosynthesis, organization, and remodeling of secondary cell wall components, primarily cellulose, hemicellulose, and lignin [14]. Advances in genomic, transcriptomic, and association-based research have begun to clarify the genetic foundation of these traits, revealing both conserved biosynthetic pathways and regulatory networks that shape the development of bast fiber (Table 1).

Bast fibers are derived from specialized cells within the phloem tissue of the outer stem cortex and have remarkable tensile strength, flexibility, and high cellulose content. The development of these fibers involves coordinated regulation of genes associated with cell division, elongation, and secondary wall deposition [114]. Transcriptomic studies have identified numerous genes involved in cellulose biosynthesis, including members of the cellulose synthase (*CesA*) gene family, as major contributors to fiber formation [115]. A genome-wide characterization of cellulose synthase (CESA) and cellulose synthase-like (CSL) gene families identified 29 *CESA/CSL* genes distributed across seven subfamilies in *C. sativa* [98]. The *CsCESA* genes are directly involved in cellulose microfibril formation, a key determinant of tensile strength, while *CSL* genes are likely to contribute to hemicellulose biosynthesis, influencing fiber flexibility and structural integrity. Tissue-specific expression patterns further indicate functional specialization of these genes in stem and fiber development, highlighting their potential as targets for genetic improvement [98].

Genome-wide association studies (GWASs) have provided additional insights into the genetic factors controlling fiber characteristics. Using more than 600,000 SNP markers, researchers identified 16 quantitative trait loci linked to fiber composition traits, including cellulose-derived sugars, lignin content, and bast fiber proportion that appeared consistently across multiple growing environments. The candidate genes found within these QTL regions are associated with carbohydrate metabolism, lignin biosynthesis, and cell wall organization, highlighting the importance of carbon allocation and polymer assembly pathways in determining fiber quality [116].

The influence of environmental conditions on fiber traits further reflects underlying gene regulatory networks. When plants are exposed to heavy-metal stress (Cd and Zn), differential expression of genes encoding cellulose synthases, fasciclin-like arabinogalactan proteins (FLAs), and class III peroxidases has been observed, indicating that cell wall biosynthesis and remodeling pathways are responsive to environmental cues [117]. While this work was conducted under stressed conditions, it points to a broader principle: the genetic networks governing fiber development are not fixed but actively tuned by signals from the surrounding environment.

Phenotypic studies conducted across a wide range of hemp germplasm have demonstrated substantial genetic variation and moderate-to-high heritability for fiber-related traits, including cellulose, hemicellulose, lignin content, and bast fiber yield [116]. Together, these findings suggest that both fiber quality and quantity are amenable to genetic improvement through marker-assisted and genomic selection strategies, supported by emerging genomic resources.

Collectively, progress in functional genomics and quantitative genetics has demonstrated that fiber quality and yield in *Cannabis sativa* are governed by a web of interconnected biosynthetic pathways and regulatory networks that integrate developmental programming with the ability to respond to environmental conditions. These findings have advanced our understanding of the genetic basis of fiber-related traits and strengthened the foundation for breeding cultivars with enhanced fiber quality, yield, and adaptability across diverse growing conditions.

## 7. Genetic Basis of Seed Quality, Oil Composition, and Yield in *Cannabis sativa*

Industrial hemp seeds are a high-value agricultural product due to their nutritional composition and expanding industrial applications. The traits that define this value are protein content, oil composition, fatty acid profile, seed size, and overall yield. These are quantitatively inherited and shaped by the interplay between genetic architecture and environmental conditions [118,119]. Recent genomic and transcriptomic work is beginning to clarify the molecular basis of these traits (Table 1), pointing to coordinated regulation across storage, metabolic, and developmental pathways.

### 7.1. Seed Storage Proteins and Nutritional Quality

The nutritional profile of industrial hemp seed is largely defined by its proteins. The primary storage protein found in industrial hemp seeds is edestin, a type of globulin protein encoded by a multigene family with distinct isoforms (e.g., *CsEde1A*–*D* and *CsEde2A*–*C*) [95]. These isoforms exhibit differential expression during seed development, with type 1 being generally more abundant and type 2 being enriched in sulfur-containing amino acids, which contributes to the seed’s overall nutritional value [95].

Additional storage proteins, including 2S albumins and 7S vicilin-like proteins, are encoded by multigene families with variable copy number and expression patterns. Transcriptomic analyses studies show that edestin and 2S albumin genes are among the highest expressed genes in seed development [96]. These findings suggest that seed protein quality is not dictated by individual genes but by the coordinated expression of multiple gene families.

### 7.2. Oil Biosynthesis and Fatty Acid Composition

In parallel with protein accumulation, industrial hemp seed oil is another key quality trait, particularly due to its high proportion of polyunsaturated fatty acids (PUFAs), including linoleic and α-linolenic acids, which vary significantly among genotypes [120]. This variation reflects genetic control over lipid biosynthetic pathways operating during seed development.

Key enzymes include stearoyl-ACP desaturase (SAD) and fatty acid desaturases (FAD2 and FAD3), which regulate desaturation steps that determine fatty acid composition. These genes are highly expressed in developing seeds and are critical to triacylglycerol (TAG) biosynthesis and PUFA accumulation [97]. Genome-wide analyses have identified approximately 36 genes involved in lipid metabolism pathways, providing a foundation for functional characterization and breeding [19].

Emerging genetic manipulation strategies, including the targeted editing of desaturase genes, suggest potential for modifying oil composition, as demonstrated in related oilseed crops [121,122,123]. However, the application of such techniques in industrial hemp remains limited and is still in the early stages of exploration.

### 7.3. Seed Size, Yield and Genetic Control

Beyond compositional traits, physical traits such as seed size and overall yield are important key determinants of overall productivity and oil output. QTL mapping studies have revealed a highly polygenic architecture underlying traits such as hundred-seed weight (HSW), seed length, width, and volume, with major loci (e.g., qHSW3, qSV1, qSL1, and qSW4) explaining substantial phenotypic variation [124]. These loci represent important targets for genetic improvement of seed yield.

In biparental mapping populations, numerous QTLs for seed and agronomic traits have been identified, with several loci explaining more than 10% of phenotypic variance [119,124]. These often cluster together in specific regions of the genome, suggesting that a relatively small number of genomic regions may control multiple yield-related traits. Importantly, many seed-related QTLs appear genetically independent from plant architecture traits, indicating the potential to improve seed yield without negatively affecting plant growth [124].

### 7.4. Seed Retention and Seed Shattering

One of the major challenges in the industrial hemp grain industry is seed shattering. This significantly reduces the marketable yield and hinders mechanical harvesting [78,125,126]. The genetic basis of seed shattering has been well studied in major cereal and oilseed crops [127], yet remarkably little is known about how *Cannabis sativa* controls seed retention and dispersal [78]. The trait most likely involves developmental pathways linked to seed attachment strength and inflorescence architecture [128]. Characterizing the genes and regulatory networks underlying seed retention will be critical to improving harvest efficiency and yield stability, particularly as hemp breeding increasingly targets grain-specific and dual-purpose genotypes [129,130]. Approaches combining genome-wide association studies, QTL mapping, and functional genomics offer a powerful framework for elucidating the genetic basis of this trait and supporting the development of genotypes with enhanced seed retention.

### 7.5. Genetic Variation and Breeding Potential

In studies examining diverse hemp germplasm collections, considerable phenotypic and genomic variation has been observed in seed protein composition, oil content, fatty acid profiles, and antinutritional factors, highlighting strong heritable control over seed quality traits [120]. The convergence of multigene family regulation, lipid biosynthesis networks, and polygenic yield loci provides a comprehensive genetic framework for seed trait improvement.

These advances reveal that seed quality and yield in *C. sativa* are governed by interconnected genetic systems spanning the regulation of storage proteins, lipid metabolism, and quantitative trait architecture. Building on this understanding, marker-assisted selection and genomic prediction represent promising approaches to developing industrial hemp genotypes optimized for seed, oil, and dual-purpose production systems. Combining detailed multi-omics profiling with functional validation will be crucial in the future to linking candidate genes to phenotypic stability and enhancing nutritional and yield-related traits across diverse environments.

## 8. Genetic Basis of Disease Resistance in Industrial Hemp

As the cultivation of industrial hemp expands across diverse agroecological regions, disease resistance has emerged as a critical target for genetic improvement [131,132]. Unlike major row crops, industrial hemp has historically lacked systematic disease resistance breeding due to regulatory constraints that limited germplasm exchange and genetic investigation.

Recent genomic work, however, is beginning to fill that gap, establishing a clear genetic basis for resistance to powdery mildew and laying the groundwork for incorporating resistance traits into modern breeding programs. In many crop species, resistance is mediated through resistance (R) genes that recognize pathogen-derived effectors and activate immune responses [133,134]. Emerging evidence suggests that similar mechanisms operate in *C. sativa*, particularly in resistance against powdery mildew, one of the most economically damaging diseases affecting industrial hemp production. A major advance was the identification of PM1, the first mapped resistance locus conferring complete resistance to powdery mildew in industrial hemp. Using high-density SNP mapping in segregating populations, PM1 was localized to a defined genomic region and demonstrated dominant inheritance of resistance. Importantly, PM1-mediated resistance was stable across environments and genetic backgrounds, indicating strong potential for breeding programs. The development of tightly linked molecular markers further enabled marker-assisted selection (MAS), representing the first practical genetic tool for disease resistance improvement in industrial hemp [135]. Beyond its applied value, PM1 established that canonical R gene-mediated immunity operates in *C. sativa*, shifting disease resistance research from phenotypic observation to mechanistic genetics [136]. This finding provided a conceptual framework for identifying additional resistance loci and applying comparative plant immunity models widely used in other crop systems.

A second resistance locus, PM2, has been identified, demonstrating that powdery mildew resistance in industrial hemp is genetically heterogeneous rather than monogenic. Unlike PM1, PM2 mediates resistance through suppression of fungal penetration and induction of a localized hypersensitive response, indicating a distinct defense mechanism. The coexistence of these loci suggests that multiple immune pathways contribute to resistance against *G. ambrosiae* [137]. Together, PM1 and PM2 constitute the first genetically defined framework for disease resistance in industrial hemp, one that is both tractable and mechanistically diverse. These loci enable marker-assisted breeding and offer a concrete entry point for integrating disease resistance into elite genotypes.

While powdery mildew represents a well-characterized disease resistance system in *Cannabis*, transcriptomic studies indicate that broader immune networks are activated in response to fungal pathogens. The dual RNA seq analysis of the interaction between *Cannabis sativa* plants and the pathogenic fungus *Sclerotinia sclerotiorum* has highlighted how infection leads to the complete reprogramming of the plant’s immune responses, defense mechanisms, and stress responses on a systems level [87]. This points to the intricate nature of the transcriptional responses involved in fungal resistance apart from just loci of resistance.

Despite these advances, resistance to many economically important pathogens remains largely unexplored. Fine mapping, gene cloning, functional validation, and the application of transcriptomic and genome-editing tools will be essential to converting these loci into durable, deployable resistance strategies and for building a broader understanding of how immunity operates in *C. sativa* as a species.

## 9. Genetic Basis of Abiotic Stress Tolerance in Industrial Hemp

As *Cannabis sativa* is increasingly cultivated across diverse environments characterized by drought, salinity, high temperature and alkaline soils, interest in the genetic basis of abiotic stress tolerance has grown considerably. Unlike traits, which can sometimes be linked to discrete loci, abiotic stress tolerance in industrial hemp is fundamentally a quantitative trait controlled by coordinated transcriptional, hormonal, metabolic responses and environmental conditions. Recent transcriptomic studies have started to map this molecular complexity in meaningful ways.

In industrial hemp, drought stress has been extensively studied at molecular level [100,138,139,140]. Genome-wide RNA sequencing under water deficit conditions has identified more than 1200 differentially expressed genes, including transcription factors from the NAC and B3 families, as well as genes involved in abscisic acid (ABA) signaling, cell wall modification, and reactive oxygen species (ROS) scavenging [100]. These results indicate that drought tolerance involves broad and coordinated transcriptional reprogramming that integrates hormonal signaling with cellular protection and water use regulation.

Studies integrating physiological and transcriptomic analyses have shown that drought response is not limited to stress signaling alone but also involves reconfiguration of primary metabolism. Rather than simply shutting down in response to stress, industrial hemp maintains growth stress balance by adjusting its carbohydrate metabolism, nitrogen utilization, and key hormone signaling pathways, including those involving ABA, auxin, and cytokinin [139]. This difference between active metabolic adjustment and passive stress suppression has important implications for how tolerance traits might be selected and improved.

Similar regulatory complexity is evident under saline and alkaline conditions. Under saline–alkaline conditions, genes involved in hormone signaling, phenylpropanoid biosynthesis, and carbon nitrogen metabolism are significantly enriched, indicating that ionic and pH stress tolerance requires coordinated metabolic and structural adaptation [141]. Suppression of photosynthetic processes combined with activation of antioxidant and detoxification pathways further highlights the dynamic nature of stress responses in *Cannabis* [142].

At a more targeted level, genome-wide analysis of the WRKY transcription factor family has helped identify key regulatory nodes within these stress networks. Multiple *CsWRKY* genes were found to be consistently induced by drought, salt, and cold stresses, with Group III WRKYs showing particularly broad and robust responsiveness across stress types [101]. Apart from the WRKY transcription factors, the genome-wide analysis of the GATA transcription factors in *Cannabis sativa* has been used for identifying lineage-specific expansion and response to stress, especially cold and salt stress during germination, indicating that these transcription factors may play regulatory roles in abiotic stress responses [143]. These transcription factors are strong candidates for central regulatory roles in coordinating abiotic stress-responsive gene expression in industrial hemp.

Moreover, the genome-wide identification and expression studies of the MYB transcription factor family indicate that MYB transcription factors exhibit dynamic regulatory activities in response to salt stress conditions in seed germination phases. Thus, MYB transcription factors also seem to contribute to abiotic stress tolerance at an early developmental phase [102]. Apart from the regulatory role of MYB transcription factors, genome-wide analysis of TIFY transcription factor genes in *Cannabis sativa* shows their involvement in alkali stress response during seed development. Additionally, there is a possibility of their involvement in cannabinoid metabolism. These findings suggest that TIFY transcription factors regulate the jasmonate signaling pathway and may act as a link between stress responses and secondary-metabolite synthesis [144].

Collectively, current evidence indicates that abiotic stress tolerance in *C. sativa* is governed by an interconnected regulatory system involving transcriptional reprogramming, hormone signaling, metabolic flexibility, and oxidative stress management. Even though the functional validation of candidate genes is limited, available genomic and transcriptomic resources have significantly improved our understanding of the genetic basis of stress adaptation. This provides a valuable framework for the genetic improvement of industrial hemp for enhanced environmental resilience.

## 10. Functional Genomics and Genome Editing

### 10.1. The Functional Validation Gap

Several candidate genes in *Cannabis sativa* associated with traits such as cannabinoid biosynthesis, sex determination, fiber development, seed quality, disease resistance, and abiotic stress tolerance have been identified through expanding genomic resources. However, the biological functions of most candidate genes are poorly characterized. For practical crop improvement in *Cannabis sativa*, functional validation serves as a bridge between genomic discovery and breeding applications. Over the past decade, research into the genetic editing and targeted engineering of *Cannabis sativa* has accelerated considerably (Table 2) but remains in its early stages relative to major crops such as rice or maize. The *Cannabis sativa* genome has long been challenging to genetically manipulate due to recalcitrance in tissue culture and transformation, limiting functional validation of genes and targeted trait improvement [145]. However, recent studies have begun to unlock both transient and stable gene manipulation systems that lay the groundwork for future breeding and trait engineering in industrial hemp [26,41,146].

### 10.2. Transient Manipulation Toolkits for Rapid Functional Screening

One of the recent breakthroughs in the field of functional genomics in *Cannabis sativa* is related to the creation of a transient reverse genetic technique based on virus-induced gene silencing (VIGS) [152]. Using a viral vector based on Cotton leaf crumple virus, successful suppression of reporter genes, including phytoene desaturase (PDS) and magnesium chelatase subunit I (ChlI) genes, was achieved. As a result, they observed a photobleaching phenotype and a reduction of approximately 70–73% in target gene transcripts [152]. This has been further expanded using Tobacco Rattle Virus (TRV) vectors as a rapid reverse genetics tool in *Cannabis sativa*, enabling efficient gene silencing in multiple tissues and allowing for the functional screening of candidate genes without stable transformation. TRV-mediated silencing successfully reduced transcript levels of target genes, providing a fast and reliable system for validating gene function in metabolic and developmental pathways [153].

Building on VIGS, *Cannabis sativa* transient expression systems were further optimized through development of an Agrobacterium-mediated agroinfiltration platform. This platform enables both transient gene expression and gene silencing across industrial hemp tissues. Using hairpin RNAi constructs targeting *CsPDS*, they observed efficient silencing and albino phenotypes in mature leaves and reproductive tissues, with transcript levels below 20% of controls. Such agroinfiltration systems are crucial to rapidly testing gene function, overexpression, and knockdown in different tissues without stable integration [155]. Transient overexpression has also successfully validated complex metabolic fluxes. A high-efficiency transient transformation system was developed in the Cheungsam genotype to overexpress CsCBDAS2, a key enzyme in the cannabinoid biosynthesis pathway. This approach resulted in coordinated upregulation of downstream cannabinoid biosynthetic genes and demonstrated the ability to functionally validate and manipulate metabolic pathways in *Cannabis* without stable transformation. Such transient overexpression systems complement VIGS, agroinfiltration, and TRV-mediated silencing by providing a flexible tool for the rapid functional analysis and metabolic engineering of target genes [105].

Although powerful for rapid gene characterization, these transient systems do not produce heritable modifications and are therefore limited to functional validation rather than direct cultivar development.

### 10.3. Stable Transformation and CRISPR-Based Genome Editing

The ultimate goal of functional genomics is the generation of stable and heritable genetic modifications. This was advanced by the first successful stable gene editing in industrial hemp using *Agrobacterium*-mediated transformation combined with CRISPR/Cas9. By optimizing explant choice and culture conditions, including the use of a developmental regulator chimera to enhance regeneration, the study generated transgenic *C. sativa* plants with targeted knockout of the phytoene desaturase gene (*CsPDS1*). Edited seedlings exhibited the expected albino phenotype, and T-DNA integration was confirmed in the *Cannabis* genome, validating both transformation and editing in a stable context. The work represents the first proof of concept for CRISPR/Cas9-based editing in industrial hemp and provides a foundation for future functional genomics and trait improvement [146]. Beyond gene knockouts, CRISPR-based technologies offer opportunities for engineering cannabinoid biosynthesis, modifying flowering behavior, manipulating sex expression, improving disease resistance, and enhancing stress tolerance.

Despite these advances, transformation and regeneration remain the major bottlenecks limiting widespread genome editing in *Cannabis*. Transformation efficiencies remain highly genotype-dependent, and many elite cultivars exhibit poor regeneration responses. An improved transformation protocol using a rapid Agrobacterium-mediated transformation method has been described: it produced stably transformed *C. sativa* plants from various explants (hypocotyls, cotyledons, and meristems) and achieved higher regeneration and transformation rates than earlier attempts. This methodology not only provides a route for transgene insertion but also enhances the potential for targeted editing using CRISPR/Cas systems by increasing the pool of transformable tissues [156].

In parallel, genotype-independent de novo regeneration systems are emerging, enhancing shoot organogenesis from explants across diverse industrial hemp cultivars without genotype limitations [157]. Scalable regeneration is arguably the rate-limiting step in industrial hemp transformation, and progress here has system-wide implications for how reliably genome editing can be applied across breeding germplasm.

### 10.4. Bottlenecks in Genome Editing and Functional Genomics

However, despite rapid advances in both transient and stable gene manipulation systems, *Cannabis sativa* still lags behind major model and crop species in the implementation of advanced genome engineering technologies. This gap is primarily due to a combination of biological and technical constraints. A key limitation is the strong genotype-dependent recalcitrance to tissue culture and regeneration, where many elite industrial hemp cultivars show poor callus induction and low shoot regeneration efficiency [158,159]. These limitations directly reduce the recovery of stable edited lines and hinder scalable genome-editing applications. In addition, low and inconsistent transformation efficiency, particularly in Agrobacterium-mediated systems [146,156], further restricts reproducibility across genotypes and laboratories, preventing the establishment of standardized editing pipelines comparable to those in model crops.

Another limiting factor is the incomplete functional annotation of *Cannabis* genomes and the lack of well-resolved gene regulatory networks for complex traits such as cannabinoid biosynthesis, sex determination, and stress adaptation [21,24]. Most candidate genes identified through these approaches remain experimentally unvalidated [147,160,161], creating a significant gap between genomic prediction and functional application.

### 10.5. Future Directions in Cannabis Genome Engineering

Current gene editing primarily relies on first-generation CRISPR/Cas9 approaches, while advanced technologies such as multiplex genome editing, base editing, prime editing, and transgene-free editing have not yet been widely implemented in *Cannabis* [162,163,164]. This limited implementation is largely constrained by regeneration inefficiency and transformation bottlenecks, as well as the absence of genotype-independent delivery systems. Further limitations are caused by the regulatory uncertainty surrounding genetically edited *Cannabis* [165].

Collectively, the development of VIGS, viral silencing systems, agroinfiltration platforms, transient overexpression tools, and CRISPR/Cas9-based genome editing represents a rapid evolution of functional genomics in *C. sativa* [146,152]. Through these advances, *Cannabis* research has been shifting from descriptive genomics toward mechanistic gene function analysis and targeted metabolic engineering. Together, they mark a transition from simple observing genomic patterns to functional and engineering-based biology. This opens new opportunities for the improvement in cannabinoid content, disease resistance, seed quality, and stress tolerance.

Looking ahead, the next phase of *Cannabis* functional genomics will require integration of next-generation genome engineering strategies with improved regeneration and transformation platforms. Future research should prioritize the adoption of multiplex genome-editing systems, enabling simultaneous manipulation of cannabinoid biosynthetic networks, regulatory transcription factors, and developmental genes [166,167]. Similarly, base editing [163,168] and prime editing [164,169] technologies offer the ability to introduce precise nucleotide changes without double-strand breaks. This is highly valuable for fine-tuning enzyme activity within cannabinoid and terpene pathways. These approaches would allow for allele-level engineering rather than complete gene disruption, enabling more predictable metabolic outcomes.

In addition, transgene-free genome-editing strategies, such as RNP-based delivery systems and DNA-free CRISPR approaches [162,170], will be essential to generating edited lines that are more acceptable for regulatory approval and commercial deployment. Integration of such systems with improved regeneration protocols could significantly accelerate the production of edited elite cultivars.

The future of *Cannabis sativa* improvement will depend on the integration of multi-omics-driven gene discovery with high-throughput functional validation and genome engineering platforms. Establishing genotype-independent transformation systems, improving regeneration efficiency, and developing standardized editing pipelines will represent a major step towards bridging the current gap between genomic knowledge and applied breeding. Such advances will enable a shift from gene discovery to programmable genome engineering, accelerating the development of industrial hemp cultivars with optimized cannabinoid profiles, enhanced stress resilience, and improved agronomic performance.

## 11. Molecular Breeding and Genetic Improvement Strategies

The integration of genomic resources into breeding pipelines has reshaped crop improvement across many species [171,172,173,174], and similar approaches are increasingly being applied to industrial hemp [175,176]. For much of its cultivated history, industrial hemp breeding operated on phenotypic selection alone, a process that is inherently slow, labor-intensive, and inefficient for complex traits influenced by numerous loci and strong genotype-by-environment interactions [177,178]. The emergence of high-quality reference genomes, pangenomic resources, genome-wide markers, and trait-mapping studies has created opportunities to transition from phenotype-driven selection toward predictive, genome-informed breeding strategies [21,27,58].

The modern molecular breeding pipeline begins with the identification of genomic regions associated with target traits through linkage mapping, genome-wide association studies (GWASs), transcriptomics, and pangenomic analyses [21,106,113]. These approaches generate candidate loci controlling economically important traits such as cannabinoid composition, flowering time, sex determination, disease resistance, fiber quality, seed oil composition, and abiotic stress tolerance [75,116,179]. Functional validation using transient expression systems, gene silencing technologies, and genome editing subsequently provides biological evidence linking genotype to phenotype [106,152]. Once validated, these loci can be converted into molecular markers that support selection within breeding populations, thereby creating a direct pathway from genomic discovery to cultivar development.

Marker-assisted selection (MAS) remains one of the earliest applications of molecular breeding in industrial hemp breeding. By using tightly linked markers to identify favorable alleles at early developmental stages, breeders can substantially reduce reliance on labor-intensive phenotyping and accelerate breeding cycles. Marker systems associated with cannabinoid profiles, sex determination, and powdery mildew resistance illustrate the potential of MAS for traits controlled by major-effect loci [72,175,180]. However, MAS is most effective for traits controlled by a small number of loci with large effects, and its utility is limited for highly polygenic traits [181]. Moreover, the development and validation of minimal SNP genotyping arrays in *Cannabis sativa* have enhanced the ability to differentiate among various cultivars and germplasms at relatively affordable costs [148]. The development of SNP-based genotyping platforms and Kompetitive Allele Specific PCR (KASP) markers has further enhanced the efficiency of germplasm characterization, cultivar identification, and marker-assisted breeding [182].

To overcome these limitations, genomic selection (GS) has emerged as a promising strategy for improving complex traits controlled by many small-effect loci [183,184]. Unlike MAS, GS uses genome-wide marker information to predict the breeding values of individuals, allowing selection without direct phenotyping. This approach captures both major and minor genetic effects and enables selection prior to phenotypic evaluation. In industrial hemp, GS holds significant promises for traits such as fiber yield, seed yield, and stress tolerance, where traditional selection is challenging. Because many commercially important industrial hemp traits exhibit complex genetic architectures and substantial environmental influence, GS can accelerate breeding far faster than traditional methods. While still emerging in industrial hemp, recent studies have demonstrated the feasibility of GS and related predictive approaches for traits such as flowering time, biomass accumulation, and cannabinoid composition [75,161]. Nevertheless, successful implementation requires large training populations, accurate phenotypic datasets, robust statistical models, and extensive marker coverage across diverse germplasm [185].

Developments in machine learning and artificial intelligence further expand the predictive capabilities of molecular breeding [186,187]. Multi-trait prediction models that integrate genomic, phenotypic, and environmental data have demonstrated improved accuracy compared with traditional statistical approaches [187]. As multi-omics datasets become increasingly available, integrating genomic, transcriptomic, metabolomic, and environmental information will enable more precise prediction of complex phenotypes and improve selection efficiency across diverse environments.

Molecular breeding strategies are also increasingly being integrated with speed breeding and controlled-environment agriculture [188,189,190]. By accelerating generation turnover, speed breeding enables more rapid population advancement and increases the efficiency of both MAS and GS [191,192]. When combined with genomic prediction and high-throughput phenotyping data, these methods create a self-improving breeding cycle where genomic data guide selection, phenotyping validates predictions, and new data continuously improve predictive models [193,194]. Such approaches have the potential to substantially reduce the time required to develop elite genotypes [189,193,194].

Before molecular breeding can be routinely implemented across industrial hemp breeding programs, several significant challenges still need to be overcome. Compared with major crops, *Cannabis* breeding has only recently gained access to high-quality reference genomes and large-scale genotyping resources [20,22,64,147,182]. Many economically important traits exhibit complex genetic architectures and strong genotype-by-environment interactions that are poorly understood [26]. In addition, limited access to diverse public germplasm collections, fragmented breeding efforts, inconsistent phenotyping protocols, and a lack of large multi-environment datasets have constrained the development and validation of robust molecular breeding tools [10,26]. As a result, the accuracy of marker-assisted and genomic selection approaches remains lower than that achieved in other major crop breeding systems.

**Figure 4:**
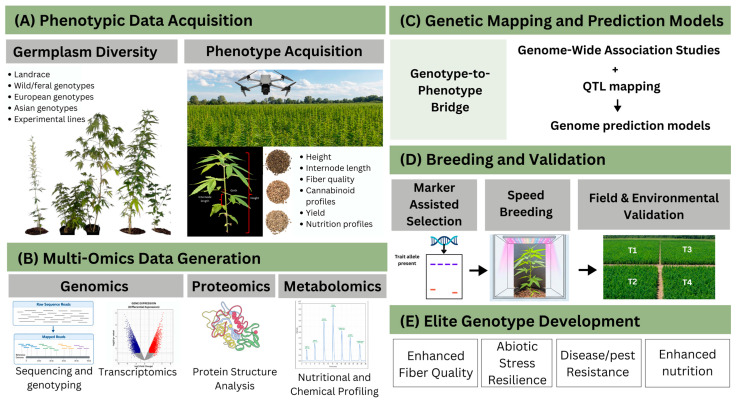
Comprehensive multi-omics integration and genomic selection framework for industrial hemp (*Cannabis sativa* L.) improvement. The workflow is organized into five sequential phases, progressing from germplasm characterization to elite cultivar development. (**A**) Phenotypic data acquisition: Evaluation of diverse germplasm, including landraces, wild/feral populations, and European and Asian genotypes, with measurement of key agronomic traits such as plant height, biomass yield, flowering time, and cannabinoid content using field trials and remote sensing approaches. (**B**) Multi-omics data generation: Integration of genomic (sequencing and genotyping), transcriptomic (gene expression), proteomic (protein profiling), and metabolomic (chemical profiling) datasets. (**C**) Genetic mapping and prediction: Identification of trait-associated loci through genome-wide association studies (GWASs) and QTL mapping, combined with genomic prediction models to support selection decisions. (**D**) Breeding and validation: Application of marker-assisted selection, speed breeding, and multi-environment field trials to validate target traits. (**E**) Elite genotype development: Development of improved cultivars with enhanced fiber quality, abiotic stress tolerance, disease and pest resistance, and improved nutritional properties. The figure was assembled in Canva with select illustrative elements generated using the DeepAI tool.

Future progress will require the integration of pangenomics, functional genomics, high-throughput phenotyping, environmental data, and advanced predictive modeling into unified breeding frameworks (Figure 4). The development of large, well-phenotyped populations across diverse environments will be essential to improving genomic prediction accuracy and identifying stable marker–trait associations. Although still in its early stages in *Cannabis sativa*, controlled-environment cultivation systems and photoperiod manipulation strategies are already being applied to enable rapid generation cycling and accelerated breeding through speed breeding approaches [195,196]. These approaches have the potential to significantly shorten breeding cycles by enabling multiple generations per year, thereby increasing selection intensity and accelerating the fixation of favorable alleles. However, their efficiency in *Cannabis* remains genotype- and environment-dependent and requires further optimization across diverse industrial hemp germplasm [195]. Furthermore, combining genomic selection with speed breeding, controlled-environment agriculture, and genome-editing technologies will enable more rapid deployment of favorable alleles and accelerate genetic gain. Emerging machine learning approaches that integrate genomic, transcriptomic, metabolomic, and environmental datasets are expected to improve prediction of complex traits and support data-driven breeding decisions [197,198,199].

Looking ahead, the future of *Cannabis* molecular breeding will likely move toward integrated genome-to-phenome platforms that combine pangenome-informed allele discovery, functional validation of candidate genes, genomic prediction modeling, and targeted genome engineering. As advanced tools become available, including genotype-independent transformation systems, transgene-free genome editing, multiplex editing, and precision breeding technologies, direct manipulation of complex genetic networks that control cannabinoid biosynthesis, flowering time, stress adaptation, fiber quality, and seed composition will be achievable. Together, these integrated pipelines have the potential to transform *Cannabis* improvement from a largely selection-based method to a more predictive, engineering-driven discipline, accelerating the development of elite cultivars optimized for fiber, grain, cannabinoid, and resilience to changing climates.

## 12. Challenges in Industrial Hemp Genetic Research

Genetic research in *Cannabis sativa* faces a combination of challenges that is, in some respects, unique among crop species. Genomic complexity, an outcrossing reproductive system, decades of regulatory restriction, and the relative youth of organized breeding programs have all left their mark on the field. Where major crops benefit from deep genetic resources, well-validated marker platforms, and generations of systematic breeding support, industrial hemp genomics must contend with high repetitive DNA content, incomplete reference assemblies, complex recombination landscapes, and persistent difficulties in trait mapping [46,58,200]. Addressing these limitations is essential to translating genomic knowledge into improved fiber, grain, cannabinoid, and climate-resilient cultivars.

At the genomic level, one of the most persistent challenges is the complexity of the *Cannabis* genome itself. Even though recent long-read sequencing technologies have significantly improved assembly quality, the genome remains difficult to characterize because of extensive repetitive sequences. Early genomic analyses revealed that a large fraction of *C. sativa* DNA comprises retrotransposons, particularly LTR/Copia and LTR/Gypsy elements, along with low-complexity sequences, which vary greatly among genotypes and contribute to assembly fragmentation and structural variation across genotypes. These repetitive elements hinder short-read assembly algorithms and complicate the placement of markers in structurally complex regions [52]. In addition, pangenomic studies have demonstrated that a single reference genome captures only a fraction of the species-wide genetic diversity. Presence–absence variation, copy number variation, and large structural rearrangements contribute significantly to trait variation but are frequently absent from traditional reference assemblies. As a result, reliance on a single reference genome can limit the identification of important alleles associated with agronomic performance [20].

These genomic challenges are further amplified at the population level by the highly heterozygous, outcrossing nature of *C. sativa* [17,201]. High heterozygosity complicates both genome assembly and genetic mapping, and assembly algorithms may collapse divergent alleles into consensus sequences or incorrectly partition haplotypes into separate contigs, leading to errors in gene annotation and inaccurate representation of sequence variation [202,203]. At the population level, heterozygosity generates linkage disequilibrium patterns that sit awkwardly within standard GWAS and QTL analysis frameworks, adding statistical complexity to an already difficult mapping environment [27].

Unlike many crop species, industrial hemp is dioecious with heteromorphic sex chromosomes. Most reference genomes focus on female (XX) plants, leaving the male (XY) genome poorly represented. This asymmetric representation makes it difficult to identify male-specific sequences and sex-determining factors, exacerbating difficulties in mapping sex-linked traits and designing sex identification markers [76]. Transcriptomic studies suggest that gene regulatory networks controlling male and female floral development are substantially divergent, and the absence of robust Y chromosome assemblies limits the ability to test candidate sex determination genes across genetic backgrounds [76].

Beyond genomic structure, a major bottleneck lies in incomplete genotype-to-phenotype resolution. Genome-wide association studies, QTL analyses, transcriptomics, and pangenomic investigations have identified numerous candidate genes associated with cannabinoid production, sex determination, fiber quality, seed composition, disease resistance, and abiotic stress tolerance [93,116,204]. However, relatively few of these candidates have undergone functional validation [144]. The limited availability of efficient transformation and genome-editing systems has slowed the transition from gene discovery to mechanistic understanding, creating a significant bottleneck in *Cannabis* functional genomics.

From a breeding perspective, industrial hemp also lacks standardized, high-density marker platforms broadly validated across diverse germplasm. Despite the recent development of genotyping tools like the HASCH SNP panel, industrial hemp still suffers from a lack of standardized, high-density marker platforms that are broadly validated across diverse germplasm. HASCH provides a mid-density set of 1504 SNPs suitable for linkage mapping and QTL analysis, but trait architectures for many important characteristics, including disease resistance, stress tolerance, and yield components, remain poorly resolved, in part due to limited marker density and uneven coverage across complex genomic regions [25]. Many agronomically important traits, such as disease resistance, abiotic stress tolerance, and fiber quality, are polygenic and subject to strong genotype-and-environment interactions. This complexity demands large datasets and precise phenotyping to disentangle genetic effects from environmental noise, a costly and time-intensive requirement that has slowed progress in industrial hemp relative to major cereals and horticultural crops [200,205,206].

At the application level, transformation and regeneration remain among the most significant technical barriers facing *Cannabis* biotechnology. While recent advances have demonstrated successful stable transformation and CRISPR/Cas9-mediated genome editing, transformation efficiency remains highly genotype-dependent and often difficult to reproduce across laboratories [26,45,207]. Many commercially important cultivars remain recalcitrant to regeneration, restricting the application of genome-editing technologies to a limited number of genetic backgrounds [207,208]. The development of genotype-independent regeneration systems and more efficient transformation platforms therefore represents critical importance for future research [209].

Another important limitation is the relative scarcity of large-scale, well-characterized breeding populations. Modern breeding approaches such as genomic selection depend on extensive phenotypic and genotypic datasets that capture genetic variation across diverse environments [210]. However, many industrial hemp breeding programs still lack sufficiently large training populations and standardized phenotyping protocols [28]. In addition, genotype-by-environment interactions substantially influence key traits, including cannabinoid content, flowering time, biomass accumulation, and stress tolerance, reducing the predictive accuracy of genomic models and complicating selection decisions [210].

The integration of multi-omics datasets represents another emerging challenge. Although individual studies have identified genes, metabolites, and regulatory pathways associated with important traits in industrial hemp, relatively few investigations have combined these datasets to develop comprehensive systems-level understanding [211]. Consequently, many of the molecular mechanisms linking genetic variation to phenotypic outcomes remain unresolved.

Regulatory and germplasm-related constraints further complicate *Cannabis* research. Legal restrictions in many regions continue to limit access to diverse germplasm, impede international exchange of genetic resources, and restrict multi location field evaluations [212]. These limitations have slowed the development of global breeding networks and reduced opportunities for comparative studies across genetically diverse populations. The fragmented nature of available germplasm collections has hindered the establishment of standardized reference panels comparable to those available in major crop species [28].

These challenges highlight the need for a more integrated approach to *Cannabis* research. Bridging these gaps is fundamental to realizing the full agricultural and industrial capacity of *Cannabis sativa*.

## 13. Future Perspectives

The future of industrial hemp genetic research is moving in a clear direction away from the study of individual traits and toward a systems-level view of the plant. Unlocking the full potential of *Cannabis sativa* will depend on how effectively researchers can bring together diverse biological data and translate them into practical breeding outcomes. Rather than being treated as distinct fields, genomics, transcriptomics, proteomics, and metabolomics are increasingly seen as interconnected, which can be used to examine the same underlying biology. Used together, these tools can reveal the regulatory networks that control a wide range of traits, from cannabinoid production and fiber characteristics to stress tolerance. This shift toward integrated multi-omics represents an important transition from largely empirical breeding toward a more predictive and design-oriented framework, where complex traits can be understood and manipulated with greater precision.

Future gene discovery efforts are likely to move beyond single-reference-genome analyses toward pangenomic and graph-based genomic frameworks. Recent studies have demonstrated that structural variation, presence–absence variation, and genotype-specific genomic regions contribute substantially to trait diversity within *C. sativa*. As additional genomes become available, graph genomes and pangenomic resources will provide a more complete representation of species-wide genetic diversity and improve the identification of functional alleles controlling economically important traits. These resources will be particularly valuable for dissecting complex traits such as fiber quality, seed yield, stress tolerance, and cannabinoid production, which are influenced by multiple genes and regulatory pathways.

Integration of multi-omics technologies will become increasingly important for understanding the molecular basis of trait variation. While genomics identifies candidate genes, transcriptomics reveals gene regulation patterns, metabolomics characterizes biochemical outputs, and epigenomics identifies environmentally responsive modifications that influence gene expression. Incorporating these datasets together will build systems-level models that accurately describe the biological pathways linking genotype to phenotype. Such approaches have already transformed research across several major crops and are expected to play a similarly important role in industrial hemp.

Functional validation of identified candidate genes will remain central to this transition. The development of CRISPR-based genome editing, together with speed breeding systems that accelerate generation turnover, is opening new possibilities for rapid trait validation and genotype development. Future genome-editing approaches may extend beyond CRISPR/Cas9 systems to include multiplex editing, base editing, prime editing, and transgene-free editing strategies. These emerging technologies offer the potential to precisely modify cannabinoid biosynthetic pathways, improve stress resilience, alter flowering behavior, and enhance seed and fiber quality while minimizing unintended genomic changes.

Predictive approaches that integrate genomic information with advanced phenotyping technologies will play a central role in shaping the future of *Cannabis sativa* crop improvement. Genomic selection, machine learning, and artificial intelligence are expected to become important components of breeding programs, particularly for complex quantitative traits that are difficult to improve using conventional selection methods. Incorporating genomic, environmental, and phenotypic data into predictive models will assist breeders to identify superior genotypes earlier in the breeding cycle and accelerate genetic gain. Incorporating high-throughput phenotyping platforms, including drone-based imaging, hyperspectral sensing, and automated environmental monitoring systems, will significantly strengthen these predictive frameworks by generating large-scale phenotypic datasets across diverse environments. Combining genomic prediction, genome editing, and speed breeding into unified crop improvement pipelines has the potential to dramatically shorten breeding cycles and increase the efficiency with which new cultivars are developed. Similar approaches are already being implemented in several major crop species and represent a logical next step for industrial hemp improvement.

Looking further ahead, the systematic expansion and characterization of global industrial hemp germplasm will be essential. Landraces and feral populations represent reservoirs of genetic diversity that are poorly explored, and that diversity may hold solutions to challenges, particularly climate-related ones, that are not yet fully understood. Generating high-quality, long-read genome assemblies for these populations and linking them to detailed phenotypic data would provide an important resource for *Cannabis* research. High-throughput consistent phenotyping methods for evaluating complex agronomic traits such as fiber-processing efficiency, metabolite profiles, and root architecture are equally important, as they would allow data to be compared and combined across studies. Coordinated common garden experiments conducted across contrasting environments would further help separate genuine genetic effects from environmental noise, particularly for traits like flowering time and regulatory compliance, where genotype-by-environment interactions can be substantial.

Looking beyond individual technologies, the future of *Cannabis sativa* research lies in building integrated genome-to-phenotype-to-breeding pipelines. The convergence of pangenomics, multi-omics integration, genome editing, artificial intelligence, high-throughput phenotyping, and accelerated breeding strategies will redefine *C. sativa* improvement over the coming decade. Beyond scientific progress alone, regulatory constraints and fragmented international policies remain significant barriers to the advancement of industrial hemp research. Better organization between regulatory authorities and collaboration among scientists from different disciplines will be just as important. With sustained investment and genuine coordination, industrial hemp has the potential to move beyond the constraints that have historically limited it, and to establish itself as a meaningful contributor to a more sustainable, bio-based economy.

## 14. Conclusions

*Cannabis sativa* L. has rapidly emerged from being an understudied crop to a genomically characterized species of considerable economic importance in both agricultural and industrial sectors. Advances in DNA-sequencing technologies, genome assembly, and pangenomic analysis have improved our understanding of the genome architecture and revealed the high level of genetic diversity underlying key trait variation. Significant advances in molecular genetics have enabled scientists to determine the biological foundations of different traits, such as the production of cannabinoids, fiber, seed quality, disease resistance, and abiotic stress tolerance.

However, most of the traits associated with industrial hemp have been proven to be polygenic and are strongly influenced by environmental interactions. Therefore, improvements in genomics are bridging the gap between research and practical genotype development through the implementation of genomic selection and editing. Overall, continued integration of multi-omics data, expanded germplasm resources, and functional validation approaches will be essential to fully realizing the potential of *Cannabis sativa* in sustainable fiber, seed, and phytochemical production systems.

## Figures and Tables

**Table 1:** Candidate genes associated with major traits.

Trait	Candidate Genes	Mechanistic Biological Function	Breeding Targets and Applications
Cannabinoid Biosynthesis	*DXS*, *GPPS*, *OLS*, *CBDAS, THCAS*, and *NBCS* [93,94]	CBGA biosynthesis and enzymatic conversion into major cannabinoids (THC, CBD, and CBC).	Development of compliant low-THC cultivars and high-yield cannabinoid medicinal cultivars.
Seed Protein Quality	*CsEde1* (A–D) and *CsEde2* (A–C) [95,96]	Edestin storage protein synthesis and regulation of seed amino acid composition.	Improvement of protein quality, digestibility, and functional food applications.
Seed Oil Profiles	*FAD2*, *FAD3*, *KCS*, and *SAD* [19,97]	Fatty acid desaturation and elongation controlling PUFA composition and oil quality.	Optimization of nutritional oil profiles and enhanced oxidative stability.
Secondary Cell Wall Deposition and Tensile Strength	*CesA4*, *CesA7*, *CesA8*, *CsCESA*/*CSL* gene families, and *IRX* [98]	Cellulose and hemicellulose biosynthesis governing fiber strength, flexibility, and cell wall architecture.	Improvement of fiber yield, tensile strength, and textile processing quality.
Fiber Quality and Lignification	*C4H*, *CCR*, and *CAD* [99]	Lignin biosynthesis and deposition within secondary cell walls.	Reduction in lignin content and improved fiber-processing efficiency.
Flowering Time	*FT*, *CO*, and *FLD* [75]	Photoperiod-mediated regulation of floral transition and reproductive development.	Adaptation to diverse environments and development of regionally optimized cultivars.
Stress Tolerance	*DREB*, *NAC*, *WRKY*, and *MYB* [100,101,102]	Regulation of drought, salinity, and heat stress responses through transcriptional and cellular defense pathways.	Development of climate-resilient cultivars with stable productivity under abiotic stress.
Defense Traits	*NBS-LRR*, *WRKY*, and *PR* genes [103]	Pathogen recognition and activation of immune signaling pathways.	Enhancement of resistance to fungal and bacterial diseases and reduction in crop losses.

**Table 2:** Functional genomics and genome-editing tools in *Cannabis.*

Method	Application	Example of *Cannabis*-Specific Target	Advantages	Limitations
QTL Mapping	Population-based; links traits to chromosomes.	Plant height, biomass, flowering time, CBD/THC variation [119] and seed size [124].	Reliable for major-effect loci; foundational for markers.	Low resolution; slow due to outcrossing nature.
GWAS (Genome-Wide Association Study)	Population-based; maps traits across diverse germplasm.	Flowering time, architecture, cannabinoids, and stress tolerance loci (*WRKY*/*NAC*/*MYB* clusters) [100,147].	Captures natural variation; high-resolution mapping.	Requires large populations; confounded by population structure.
SNP Genotyping and Marker Development	High-throughput screening; marker development (MAS).	Cultivar identification, sexing, and chemotype panels (1.5K HASCH, 20-SNP panels) [25,148].	Early seedling sexing/chemotype prediction; cost-effectiveness.	Requires prior discovery; poor for complex polygenic traits.
RNA-seq (Transcriptomics)	Expression profiling across tissues or abiotic/biotic stressors.	Sex-linked/male-biased genes; transcripts for environmental sex plasticity [149,150].	Global expression profiles; identifies candidate pathway genes.	Correlation only; highly tissue-, time-, and condition-specific.
Protoplast Transient Expression Assays	Transient; rapid subcellular and promoter analysis.	PEG-mediated system in isolated hemp cells for functional validation [151].	Fast feedback (24–48 h); bypasses whole-plant regeneration.	Short expression window; highly sensitive to tissue age/genotype.
Virus-Induced Gene Silencing (VIGS)	Transient; rapid gene knockdown.	Silencing of *PDS* and *ChlI* for validation; cannabinoid/pigment pathways [152,153].	Fast pipeline; eliminates tissue culture/regeneration needs.	Temporary effects; variable knockdown; uneven systemic spread.
Agrobacterium-Mediated Transformation	Stable; gene introduction and trait engineering.	Transgenic calli expressing *AtNPR1* (disease) and bar (herbicide) genes [154].	Heritable genome integration; permanent transgenic lines.	Very time-consuming; bottlenecked by poor regeneration.
CRISPR/Cas9 Genome Editing	Stable; precise gene knockout or knockin.	Knockout of *CsPDS1* (albino phenotype proof of concept) [146].	High precision; definitive causal validation of gene function.	Off-target risks; limited by recalcitrant tissue regeneration.

## Data Availability

No new data were created or analyzed in this study.

## Supplementary Materials

The following supporting information can be downloaded at: https://www.mdpi.com/article/10.3390/plants15132088/s1. Table S1. Overview of Cannabis sativa Genome Assemblies and Key Assembly Metrics.

## Abbreviations

The following abbreviations are used in this manuscript:

CBD	Cannabidiol
CBDAS	Cannabidiolic Acid Synthase
CBDA	Cannabidiolic Acid
CBGA	Cannabigerolic Acid
GS	Genomic Selection
GWAS	Genome-Wide Association Study
LTR	Long Terminal Repeat
MAS	Marker-Assisted Selection
Mb	Megabase
QTL	Quantitative Trait Locus
SNP	Single Nucleotide Polymorphism
SSR	Simple Sequence Repeat
THC	Tetrahydrocannabinol
THCAS	Tetrahydrocannabinolic Acid Synthase
THCA	Tetrahydrocannabinolic Acid
TRV	Tobacco Rattle Virus
VIGS	Virus-Induced Gene Silencing
