# Supplementary material for: Genetic Advances in Cannabis sativa L.: A Review of Recent Progress and Future Directions
Source: Plants (Basel). 2026 Jul 4;15(13):2088. doi: 10.3390/plants15132088 (PMC13363910; doi:10.3390/plants15132088)
Supplement: Supplementary file 1 [file plants-15-02088-s001.zip › plants-4336798-supplementary.pdf]

Table S1. Overview of *Cannabis sativa* Genome Assemblies and Key Assembly Metrics.

	Genotype / Assembly	Accession (NCBI/NGDC)	Approx. Genome Size	Assembly Stats (N50, contigs/scaffolds)	Sex / Type	Reference / Notes
2011	Purple Kush	GCA_000230575.1	~818–843 Mb	Draft contig/scaffold	Drug-type / female	First draft genome; early sequencing efforts
2016	Chemdog91	GCA_001509995.1	~285.9 Mb	Scaffold N50 ~2 kb; 190,122 contigs	Female / drug-type	Highly fragmented Illumina GAII assembly
2016	Cannatonic	GCA_001865755.1	~585.8 Mb	Contig N50 ~128 kb; 11,110 contigs	Female	Early PacBio draft genome
2017	Pineapple Banana Bubba Kush	GCA_002090435.1	~512.2 Mb	Contig N50 ~51.8 kb; 18,355 contigs	Drug-type	Early PacBio draft assembly
2018	Finola	GCA_003417725.2	~1009.7 Mb	Chromosome-level; N50 ~370 kb	Hemp / fiber-type	Early hemp genome assembly
2018	Purple Kush	GCA_000230575.5	~891.9 Mb	Chromosome-level	Drug-type / female	High-quality PacBio assembly
2019	Jamaican Lion DASH	GCA_003660325.2	~1073.9 Mb	Contig N50 ~3.81 Mb	Female / drug-type	Long-read PacBio assembly
2019	CBDRx (cs10)	GCF_900626175.2	~875.7 Mb	Scaffold N50 ~91.9 Mb; 1,051 contigs	High-CBD / female	Chromosome-resolved genome; cannabinoid loci mapped, ONT + Illumina hybrid assembly
2020	Wild-type female (Tibet)	GCA_013030365.1	~812.5 Mb	Scaffold N50 ~83.0 Mb; Contig N50 ~0.51 Mb	Wild type	PacBio + Hi-C assembly
2020	Cannbio-2	GCA_016165845.1	~914.4 Mb	Scaffold N50 ~91.5 Mb; 8,919 contigs	Female / balanced THC-CBD	High-quality chromosome assembly
2020	Jamaican Lion DASH / derivatives	GCA_016415525.1	~999.1 Mb	Contig N50 ~3.49 Mb	Female	PacBio assembly
2020	Jamaican Lion v4	GCA_012923435.1	~876.6 Mb	Contig N50 ~3.28 Mb	Female	Annotated drug-type genome
2020	Jamaican Lion v4	GCA_013030025.1	~1008.8 Mb	Contig N50 ~1.67 Mb	Male	Male reference genome
2020	Cannatonic	GCA_001865755.1	~585.8 Mb	Contig N50 ~128 kb	Female	PacBio assembly
2020	Pineapple Banana Bubba Kush	GCA_002090435.1	~512.2 Mb	Contig N50 ~51.8 kb	Drug-type	PacBio assembly

2022	Abacus	GCA_025232715.1	~796.6 Mb	Chromosome-level; N50 ~80.6 Mb	Female / CBD-type	PacBio Sequel assembly
2023	Pink Pepper	GCA_029168945.1	~770.3 Mb	Chromosome-level; Contig N50 ~23.5 Mb	Female / hemp	Hybrid ONT + PacBio + Illumina
2023	Pink Pepper	GCF_029168945.1	~770.3 Mb	Chromosome-level; Scaffold N50 ~76.98 Mb	Female / hemp	RefSeq version; annotated genome
2025	Hawthorne OG	GCA_051911645.1	~699.7 Mb	Scaffold N50 ~76.2 Mb	Drug-type	ONT + Illumina assembly
2025	Anders CBD	GCA_051911655.1	~713.2 Mb	Scaffold N50 ~74.4 Mb	CBD-type	Draft chromosome assembly
2025	Anders CBD	GCA_051911675.1	~748.3 Mb	Scaffold N50 ~78.5 Mb	CBD-type	Improved assembly version
2025	Travis CBD	GCA_051911765.1	~738.6 Mb	Scaffold N50 ~78.0 Mb	CBD-type	Chromosome-level draft
2025	Travis CBD	GCA_051911785.1	~735.6 Mb	Scaffold N50 ~79.6 Mb	CBD-type	Improved assembly
2025	Hawthorne OG	GCA_051911705.1	~682.5 Mb	Scaffold N50 ~73.1 Mb	Drug-type	Chromosome assembly
2025	Sri Lankan	GCA_051911735.1	~646.0 Mb	Scaffold N50 ~68.3 Mb	Landrace	Low-contiguity diversity genome
2025	Sri Lankan	GCA_051911715.1	~641.8 Mb	Scaffold N50 ~66.4 Mb	Landrace	Additional assembly
2025	Punto Rojo × Cherry Pie	GCA_049725155.1	~811.3 Mb	Scaffold N50 ~87.6 Mb	Hybrid	ONT + Illumina assembly
2026	Unnamed complete genome	GCA_054642775.1	~756.0 Mb	Complete genome; N50 ~81.0 Mb	—	ONT + PacBio + Illumina
2026	Unnamed complete genome	GCA_054642815.1	~751.6 Mb	Complete genome; N50 ~79.7 Mb	—	High-quality complete assembly

Note: Data presented in Table 1 were compiled from publicly available records in the National Center for Biotechnology Information (NCBI) databases.
